# Electroacupuncture and carbamazepine for patients with trigeminal neuralgia: a randomized, controlled, 2 × 2 factorial trial

**DOI:** 10.1007/s00415-024-12433-x

**Published:** 2024-05-31

**Authors:** Rongrong Li, Jing Sun, Kaitao Luo, Ning Luo, Ruohan Sun, Feng Gao, Yiyi Wang, Yunfan Xia, Xiaoyu Li, Lifang Chen, Ruijie Ma, Xiaomei Shao, Yi Liang, Jianqiao Fang

**Affiliations:** 1 The Third Affiliated Hospital of Zhejiang Chinese Medical University, Hangzhou, China; 2 Jiaxing Hospital of Traditional Chinese Medicine, Jiaxing, China; 3 The Third Clinical Medical College of Zhejiang Chinese Medical University, 548 Binwen Road, Hangzhou, Zhejiang China; 4 Department of Neurobiology and Acupuncture Research, Key Laboratory of Acupuncture and Neurology of Zhejiang Province, 219 Moganshan Road, Hangzhou, Zhejiang China

**Keywords:** Trigeminal neuralgia, Electroacupuncture, Carbarmazepine, Factorial design, Randomized controlled trial

## Abstract

**Background:**

Trigeminal neuralgia (TN) is difficult to treat due to its severe pain intensity and recurring episodes, which significantly impact quality of life.

**Objectives:**

We aimed to assess the effectiveness of electroacupuncture (EA) in alleviating the pain intensity in TN, and to determine whether EA combined with low-dosage carbamazepine (CBZ) has a synergistic effect.

**Methods:**

A multi-centre, randomized, 2 × 2 factorial trial was conducted. Participants who met the inclusion criteria received active EA or sham EA for 60 min, three times a week for four weeks; CBZ (300 mg per day) or placebo for four weeks. The primary outcome was the change in visual analog scale (VAS) score from baseline to weeks 2, 4, 16, and 28. Secondary outcomes included quality of life and adverse events.

**Results:**

A total of 120 participants (75 females and 45 males; mean (SD) age, 58.5 (15.3) years) were included. The main effects of EA and CBZ were significant (*P* < 0.001), and there was a significant interaction was identified between the interventions (*P* = 0.041). Participants who received EA (mean difference [MD], −0.3 [95% CI, −0.40 to −0.20] at week 2; −1.6 [−1.70 to −1.50] at week 4; −1.1 [−1.31 to −0.89] at week 16; −0.8 [−1.01 to −0.59] at week 28), CBZ (MD, −0.6 [95% CI, −0.70 to −0.50] at week 2; −0.9 [−1.03 to −0.77] at week 4, −0.2 [−0.41 to 0.01] at week 16, 0.2 [−0.01 to 0.41] at week 28), and the combination of both (MD, −1.8 [95% CI, −1.90 to −1.70] at week 2; −3.7 [−3.83 to −3.57] at week 4, −3.4 [−3.61 to −3.19] at week 16, −2.9 [−3.11 to −2.69] at week 28) had a greater reduction in VAS score over the treatment phase than their respective control groups (sham EA, placebo, and sham EA plus placebo). EA-related adverse events (6/59, 10.17%) were lower than that of CBZ (15/59, 25.42%) during the whole phases.

**Conclusions:**

EA or CBZ alone are effective treatments for TN, while the combination of EA and low-dosage CBZ exerts a greater benefit. These findings in this trial demonstrate that the combination of EA and low-dosage CBZ may be clinically effective under certain circumstances.

**Trial registration:**

NCT03580317.

**Supplementary Information:**

The online version contains supplementary material available at 10.1007/s00415-024-12433-x.

## Introduction

Trigeminal neuralgia (TN) is a common neuropathic pain condition characterized by unilateral, transient, and recurrent episodes of pain confined to one or more branches of the trigeminal nerve [1, 2]. Patients with TN often experience stabbing, burning, or brief electric shock-like pain triggered by certain stimuli in specific trigger areas, such as eating, brushing, and touching [3]. The incidence of TN is estimated at 12.6 cases/100,000 person-years, and women are more frequently affected (60%) [4]. The intensity and irregularity of pain can lead to mental disorders such as anxiety, depression, and insomnia, which significantly decreased the quality of life [5].

Acupuncture, a common treatment in Traditional Chinese Medicine, has been widely used for many years to manage TN, especially in patients with drug-refractory [6–9]. Meta-analyses have shown that electroacupuncture (EA), a worldwide-used acupuncture therapy, may be more effective in alleviating pain intensity compared to carbamazepine (CBZ) or other common therapies [10–12]. However, the current evidence for acupuncture and EA in treating TN is limited due to the generally poor quality of relevant randomized controlled trials (RCTs), such as small sample sizes, improper controls, and lack of follow-up [10]. Our previous observational trial suggested that EA may be superior to low-dosage CBZ (300 mg/day) in relieving pain, improving facial-specific activities, and enhancing the quality of daily life in patients with TN [13].

CBZ, a first-line and inexpensive drug, is recommended for long-term treatment of TN according to the guidelines of the European Federation of Neurological Societies (EFNS) [14]. Under the guidelines of EFNS, the recommended therapeutic dosage range for CBZ was from 200 to 1200 mg/day. However, high dosages of CBZ (approximately 600 mg/day) can cause side effects such as somnolence, dizziness, and postural imbalance [15–17]. A real-world study of 354 patients with TN reported that oral administration of CBZ at a dosage of 600–800 mg/day resulted in a higher incidence of side effects (43.6%), including somnolence, unbalance, and dizziness [18]. Furthermore, CBZ may have a 50% failure rate for long-term (5–10 years) pain control under the guidelines of EFNS [14]. Although surgery is the recommended choice for refractory TN, complications such as trigeminal sensory disturbance, masticatory atonia, and auditory perceptual disorders were inevitable [19–22]. Therefore, there is an urgent need for high-quality clinical trial evidence.

Thus, a multi-centre randomized clinical trial with sound methodology was carried out to assess the effectiveness of EA in alleviating the pain intensity in TN, and to further determine whether EA combined with low-dosage CBZ has a synergistic effect.

## Methods

### Study design

**Fig. 1:**
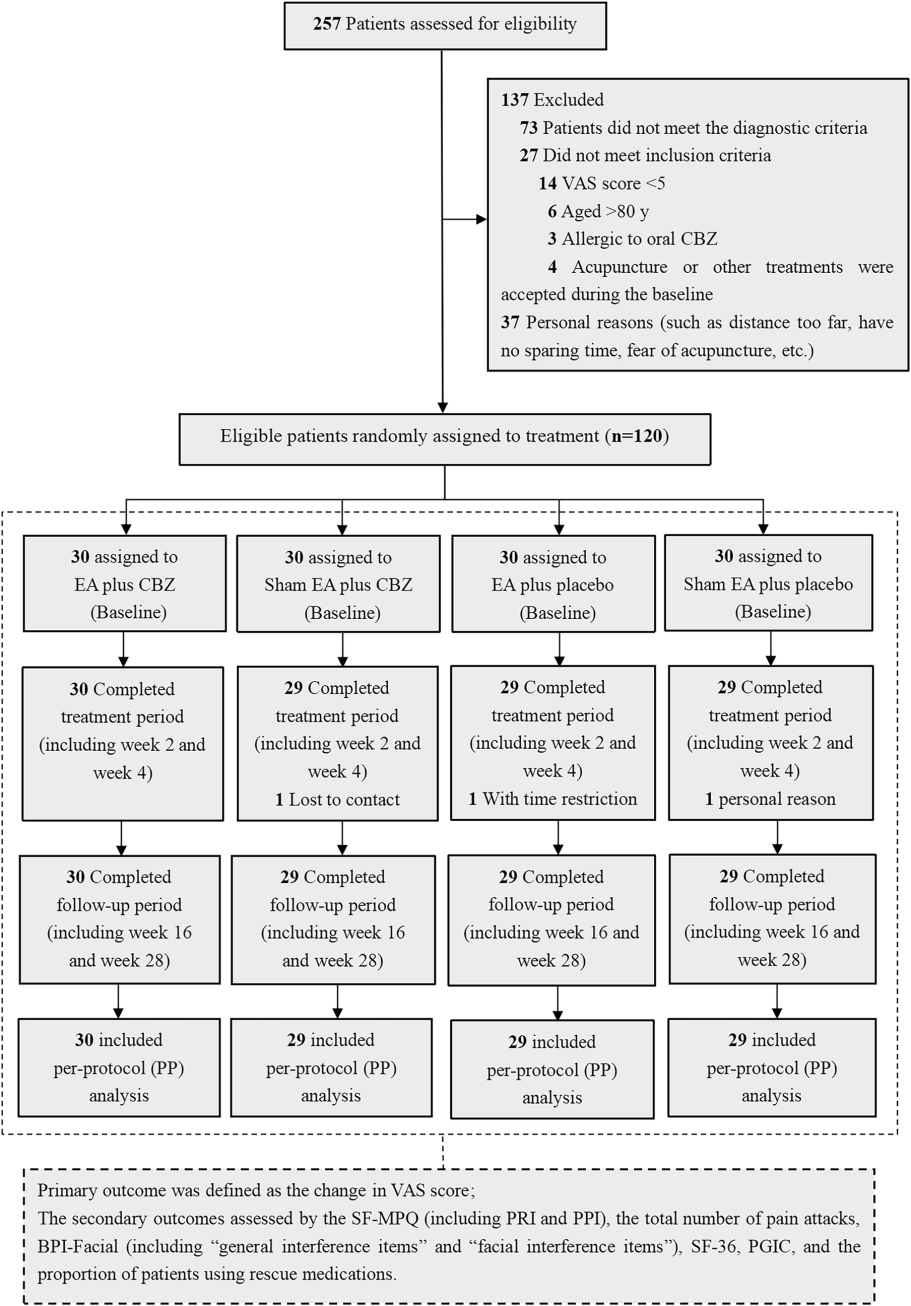
Modified CONSORT flow diagram for the trial. Abbreviations: VAS = visual analogue scale; EA = electroacupuncture; CBZ = carbamazepine; SEA = sham electroacupuncture; P = placebo; SF-MPQ = Short-Form McGill pain questionnaire; PRI: pain rating index; PPI: present pain intensity; BPI-Facial: brief pain inventory-facial scale; PGIC: patient global impression of change

A multicenter, randomized, and controlled clinical trial was conducted at two hospitals in Zhejiang Province, China (i.e., *The Third Affiliated Hospital of Zhejiang Chinese Medical University* and *Jiaxing Hospital of Traditional Chinese Medicine*), with a two-by-two factorial design. Patients were recruited from July 12, 2018, to January 31, 2021, with a total study duration of 30 weeks, including a 2-week baseline, 4-week treatment period, and 24-week follow-up period (eFigure 1). The modified CONSORT flow diagram is shown in Fig. 1, and the study protocol was previously published [23].

### Ethical considerations and trial registration

All patients signed informed consent forms before enrolling in the trial. Ethical approval was obtained from the Clinical Trial Ethics Committee of the *Third Affiliated Hospital of Zhejiang Chinese Medical University* (No. ZSLL-KY-2017-033) and *Jiaxing Hospital of Traditional Chinese Medicine* (No. 2018-JZLK-002). Furthermore, this trial was registered on *the National Institutes of Health (NIH)* at website (Clinical Trials.gov) with the trial registration number NCT03580317.

### Participants

#### Diagnostic criteria

The diagnostic criteria for TN was in accordance with the *International Classification of Headache Disorders (3rd Edition)*, which published by the Headache Classification Committee of the International Headache Society (IHS) in 2013 [24]. The details were as follows: (1) recurrent paroxysms of unilateral facial pain in the distribution(s) of one or more divisions of the trigeminal nerve, with no radiation beyond and fulfilling criteria (2) and (3); (2) pain has the following characteristics: (a) lasting from a fraction of a second to two minutes; (b) severe intensity; (c) electric shock-like, shooting, stabbing or sharp in quality; (3) precipitated by innocuous stimuli within the affected trigeminal distribution; (4) no neurological impairment; (5) other diseases, which could cause the pain, was eliminated.

#### Inclusion criteria

The inclusion criteria were as follows: (1) electric shock pain, shooting pain, or stabbing pain occurring in one or more branches of the trigeminal nerve; (2) visual analog scale (VAS) score ≥ 5 at baseline and attacks more than 3 times per day and at least 4 days per week; (3) males or females, aged between 18 and 80 years; (4) patients who were conscious, communicable, and willing to sign a written informed consent form.

#### Exclusion criteria

Participants with the following conditions were excluded: (1) epilepsy, head injury, severe heart disease, cognitive impairment, aphasia, psychiatric disorders, poorly controlled hypertension, etc.; (2) individuals diagnosed with major depressive disorder, pregnant or breastfeeding women were also excluded; (3) secondary TN (e.g., multiple sclerosis, space-occupying lesions, etc.) were also not considered for inclusion. Notably, included patients received transportation allowances and free treatment to improve compliance.

### Sample size

The sample size was estimated by calculating the mean change of VAS score, which was determined in a previous study [25] and in our preliminary experiment. The mean VAS scores in the groups of EA plus CBZ, sham EA plus CBZ, EA plus placebo, and sham EA plus placebo were 5.23, 4.45, 5.50, and 0.00, respectively, with a standard deviation of 1.6. At an alpha level of 0.05, the test efficiency (1 − β) was 0.8. A total of 120 cases were included in four groups.

### Randomization and blinding

Participants were randomly allocated by a central randomized system in a 1:1:1:1 ratio into four groups: EA plus CBZ, sham EA plus CBZ, EA plus placebo, and sham EA plus placebo. Given the specificity of acupuncture, the evaluators, data collectors, and statisticians were blinded to the allocation of acupuncture treatment, except for the acupuncturists. Each patient was treated individually to avoid communications between patients about treatment, feelings, and therapeutic effects, and to better implement blinding.

### Interventions

The location of acupoints was determined according to the People’s Republic of China National Standard, “The Name and Location of Acupoints” (GB/T 12346-2006). Additionally, all acupuncturists were licensed and experienced attending physicians. Patients were treated three times per week for four weeks, for a total of 12 EA sessions.

#### EA or sham EA

In the EA groups, needles (25 mm in length, 0.18 mm in diameter; Hwato) were inserted in *Sibai (ST2)*, *Xiaguan (ST7)*, and *Dicang (ST4)* as the main acupoints. Additional acupoints of *Tongziliao (GB1)*, *Quanliao (SI18)*, or *Jiache (ST6)* were selected to match the location of pain in the ophthalmic, maxillary, or mandibular branches, respectively. Needles (40 mm in length, 0.25 mm in diameter; Hwato, Co., Ltd., Beijing, China) were inserted bilaterally into *Hegu (LI4)* and *Waiguan (SJ5)*, with manipulation of lifting-thrusting and twirling to get stronger sensation. Besides, lesions in the ophthalmic, maxillary, or mandibular branches were treated with *ST7/GB1*, *ST7/SI18*, or *ST7/ST6,* respectively, using the HANS Acupuncture Point Nerve Stimulator (*HANS-200A Huawei Co., Ltd., Beijing, China*). Distal acupoints of *LI4* and *SJ5* were also selected. The specific acupoints, stimulation, and locations were shown in eFigure 2 and eTable 1. The frequency of EA was 2 Hz and 100 Hz alternating waves, with a treatment time of 60 min, and a current intensity ranging from 0.5 to 1 mA, depending on the individual’s needs. The duration of EA stimulation was determined based on expert experience.

In the sham EA groups, non-acupoints that were located 1 cm lateral to the same acupoints were selected. Electrodes were connected to non-acupoints near by *ST7/GB1,* or *ST7/SI18,* or *ST7/ST6* and *LI4/SJ5*. For successful blinding, the parameter settings on the instrument screen and the device were visible, while no current output was generated.

#### CBZ or placebo

CBZ or placebo was routinely administered 100 mg, three times per day, seven days per week. All aforementioned treatments were administered for four consecutive weeks.

CBZ and EA were stopped without any tapering period at end of 4-week treatment phase, but rescue medications were allowed. Participants in the sham EA plus placebo group did not receive real EA and oral CBZ, but were informed that 12 sessions of EA treatment would be provided free of charge at the end of the trial.

### Rescue medications

Participants were prohibited from receiving any other treatments besides rescue medication during all three phases of this trial, including the 2-weeks baseline. However, in accordance with the “Declaration of Helsinki”, rescue medication was allowed for patients with unbearable pain. Participants were allowed to administrate a 200 mg dosage of CBZ after meals, with a maximum daily dosage of 600 mg in this trial. The timing and dosage of all rescue medications was recorded.

As for the administration of CBZ, the rescue medication was allowed during the observed phases including baseline, treatment and follow-up phases, but the routine usage of 300 mg CBZ daily was only administrated in the treatment phase. The dosage of CBZ in rescue administration started at 200 mg, then adjusted according to individual’ s need, but limited at 600 mg daily; while routine administration (300 mg daily) was not allowed to be adjusted.

### Assessment and outcomes

Participants were informed to complete a daily pain diary, which included the following issues: (1) the daily number of pain attacks in the past 24 h (calculated by summing the number of pain attacks, including spontaneous and induced pain); (2) triggering factors; (3) the daily VAS scores, which were recorded as the average of pain intensity in the past 24 h; (4) adverse events induced by treatment (including EA and CBZ) in the past 24 h; and (5) the application of rescue medications.

#### Primary outcome

Primary outcome was defined as the change in VAS score from baseline to weeks 2, 4, 16, and 28. *The Assessment Committee of the Neuropathic Pain Special Interest Group (NeuPSIG) of the International Association for the Study of Pain (IASP)* recommends the use of the VAS to assess pain intensity in individuals with neuropathic pain, and the effects of treatment on neuropathic pain intensity in clinical trials (*level A*) [26]. The VAS score was specifically evaluated as follows: according to the pain diary, the mean VAS score in the pre-treatment phase (the past two weeks) was recorded as the baseline data; 1st and 2nd weeks were used for the week 2 data; the mean VAS score in the past 3rd and 4th weeks were used for the week 4 data. During the follow-up phases, the past 15th and 16th weeks were used for the week 16 data, and 27th and 28th weeks were used for the week 28.

#### Secondary outcomes

The secondary outcomes, included the following measures: (1) Other outcomes to assess the pain efficacy. Short-Form McGill Pain Questionnaire (SF-MPQ), which included the “pain rating index” (PRI) and “present pain intensity” (PPI), with higher scores indicating greater pain [27]. The total number of pain attacks, calculated by the pain diary, recording the total number of pain attacks in the past two weeks prior to every assessment time-point. The total number of pain attacks in the pre-treatment phase (the past 2 weeks) was recorded as the baseline data; the summing of pain attacks in 1st and 2nd weeks were recorded as the week 2 data; and 3rd and 4th weeks were recorded as the week 4 data. During the follow-up phases, the summing of pain attacks in the past 15th and 16th weeks were recorded as the week 16 data; and 27th and 28th weeks were recorded as the week 28); (2) Quality of life assessment. Brief Pain Inventory-Facial scale (BPI-Facial), which included the “general interference items” and “facial interference items”, ranging from 0 to 70, with higher scores indicating a greater impact [28]; the Short-Form 36 Questionnaire (SF-36); Patient Global Impression of Change (PGIC), with disease deterioration (0 to 3 points), stable disease (4 points), or disease improvement (5 to 7 points) since the initial baseline visit [29]. (3) The proportion of patients using rescue medications; and (4) Adverse events (AEs).

The mentioned outcomes were evaluated at baseline, week 2, week 4, follow-up week 16, and follow-up week 28.

#### Blinding and compliance

Participants’ responses to trust and expectation questionnaires for acupuncture were completed at baseline. All participants were asked to guess the treatment which they have received at the end of the trial and the answers were recorded, which presented as the distribution of guesses of treatment received. Any adverse events, including those caused by EA or CBZ, were managed and recorded appropriately. Furthermore, treatment compliance was also recorded.

### Statistical analysis

All data from this trial were analyzed by the “Clinical Data Center of Zhejiang Provincial Hospital of Chinese Medicine” using SPSS version 25.0. Only patients who completed the final evaluation were included in the preliminary analysis. Continuous variables that followed a normal or approximately normal distribution were reported as the mean *(SD)*, while those that did not are reported as the medians *(P25, P75)*. Percentages and frequencies were calculated for the categorical variables. Analysis of variance *(ANOVA)* was used for normally distributed continuous variables, whereas the *Kruskal–Wallis H* test was used for data with non-normal distributions. The *chi-square* test was used to evaluate the categorical data. Importantly, a factorial trial focused on the main effects of the two treatments and their interaction was performed [30]. Therefore, a repeated-measures ANOVA with factorial design was used to assess the efficacy of EA combined with CBZ, and the *Sidak* test was used for multiple comparisons between groups. The proportions of participants using rescue medications were compared between groups using the *chi-square* test or *Fisher’s exact* test. Statistically significant differences were indicated by a two-sided *P* < 0.05.

## Results

### Participant flow

A total of 257 patients were screened for eligibility at two centers from July 2018 to January 2021. Among them, 120 eligible patients were randomly assigned into four groups of 30 individuals each. Treatment and follow-up visits for all patients were completed in August 2021. Three participants withdrew at baseline for the following reasons: one lost contact (3.33%), one dropped out due to the travel distance (3.33%), and another dropped out due to poor efficacy (3.33%). The remaining 117 (97.50%) patients with TN completed the intervention and follow-up assessments. The flow diagram of this study is shown in Fig. 1.

### Baseline characteristics

The baseline characteristics of the participants were shown in Table 1. The incidence of pain along the maxillary and mandibular branches was higher than that along the ophthalmic branch, consistent with previous studies [31]. Notably, the majority of participants (78 out of 120, 65%) suffered from emotional disorders such as insomnia, irritability, anxiety, and depression. Only 17.6% of the included patients were satisfied with the treatments that they received before participating in this trial, and 77 out of the 120 patients (64.2%) remained uncertain about the effectiveness of acupuncture for TN.

**Table 1:** Baseline characteristics

Characteristics	Group				All patients (n = 120)	Statistics value	*P* value^a, b^
	EA + CBZ (n = 30)	SEA + CBZ (n = 30)	EA + P (n = 30)	SEA + P (n = 30)			
Sex, n (%) ^b^						*χ*^2^ = 3.520	0.318
Male	15 (50.0)	11 (36.7)	8 (26.7)	11 (36.7)	45 (37.5)		
Female	15 (50.0)	19 (63.3)	22 (73.3)	19 (63.3)	75 (62.5)		
Age, mean (SD), years^a^	58.7 (13.1)	54.2 (18.8)	60.0 (13.9)	60.9 (14.9)	58.5 (15.3)	*F* = 1.134	0.338
Duration of illness, M (P25, P75), Mon ^a^	25.5 (11.3,48.0)	12.0 (2.0,36.0)	24.0 (2.0,61.3)	33.0 (11.0,60.0)	24.0 (7.0,48.0)	*χ*^2^ = 4.667	0.198
Pattern of pain attack, n (%)^b^						*χ*^2^ = 0.001	1.000
Purely paroxysmal	29	28	28	29	114		
With CCP	1	2	2	1	6		
Side of pain attack, n (%)^b^						*χ*^2^ = 0.733	0.866
Left	12 (40.0)	11 (36.7)	9 (30.0)	10 (33.3)	42 (35.0)		
Right	18 (60.0)	19 (63.3)	21 (70.0)	20 (66.7)	78 (65.0)		
Localization of pain attack, n (%)^b^						*χ*^2^ = 9.154	0.171
The 1st trigeminal division (Ophthalmic branch)	9 (30.0)	5 (16.7)	12 (40.0)	6 (20.0)	32 (26.7)		
The 2nd trigeminal division (Maxillary branch)	8 (26.7)	15 (50.0)	6 (20.0)	10 (33.3)	39 (32.5)		
The 3rd trigeminal division (Mandibular branch)	13 (43.3)	10 (33.3)	12 (40.0)	14 (46.7)	49(40.8)		
Emotional impact, n (%)^b, c^						*χ*^2^ = 1.905	0.592
Yes	19 (63.3)	17 (56.7)	22 (73.3)	20 (66.7)	78 (65.0)		
No	11 (36.7)	13 (43.3)	8 (26.7)	10 (33.3)	42 (35.0)		
Previous treatment for TN, n (%)^b,d^						*χ*^2^ = 4.907	0.842
Acupuncture	14 (66.7)	12 (75.0)	15 (65.2)	18 (72.0)	59 (69.4)		
Analgesics	3 (14.3)	1 (6.3)	1 (4.3)	2 (8.0)	7 (8.2)		
Acupuncture plus analgesic	4 (19.0)	2 (12.5)	6 (26.1)	3 (12.0)	15 (17.6)		
Others (including surgery, etc.)	0 (0.0)	1 (6.3)	1 (4.3)	2 (8.0)	4 (4.7)		
Satisfaction with previous treatment, n (%)^b^	5 (23.8)	2 (12.5)	3 (13.0)	5 (20.0)	15 (17.9)	*χ*^2^ = 1.271	0.736
Expectations of acupuncture for diseases, n (%)^b^						*χ*^2^ = 4.593	0.597
Effective	6 (20.0)	7 (23.3)	10 (33.3)	7 (23.3)	30 (25.0)		
Invalid	0 (0.0)	0 (0.0)	0 (0.0)	1 (3.3)	1 (0.8)		
Uncertain	24 (80.0)	23 (76.7)	20 (66.7)	22 (73.3)	89 (74.2)		
Expectations of acupuncture for TN, n (%)^b^						*χ*^2^ = 8.364	0.213
Effective	8 (26.7)	8 (26.7)	11 (36.7)	15 (50.0)	42 (35.0)		
Invalid	0 (0.0)	0 (0.0)	0 (0.0)	1 (3.3)	1 (0.8)		
Uncertain	22 (73.3)	22 (73.3)	19 (63.3)	14 (46.7)	77 (64.2)		
The mean of VAS score^e^	6.3 (1.1)	6.5 (1.2)	6.3 (1.0)	6.0 (1.1)	NA	*F* = 0.038	0.846
The total number of pain attacks^f^	199.8 (133.6)	230.5 (210.0)	161.8 (79.5)	225.0 (211.5)	NA	*F* = 2.067	0.153

^a^Continuous data are expressed as mean *(SD)* and examined using *t* test

^b^Categorical data were examined using *Chi-square* or *Fisher Exact* test

^c^Patients with TN accompanied by emotional disorders, such as anxiety and depression

^d^Previous treatment for TN from onset to 1 month pre-enrollment

^e^The mean daily of VAS score was recorded as the average of pain intensity in the past 24 h

^f^The total number of pain attacks was calculated by summing the number of pain attacks in the past two weeks prior to the every assessment time-point

Abbreviations: *TN* trigeminal neuralgia, *SD* standard deviation; *M* medium, *CCP* concomitant continuous pain, *NA* not applicable, *EA + CBZ* electroacupuncture plus carbarmazepine (at the dosage of 300 mg/day), *SEA + CBZ* sham electroacupuncture plus carbarmazepine (at the dosage of 300 mg/day), *EA + P* electroacupuncture plus placebo, *SEA + P* sham electroacupuncture plus placebo

### Primary outcome

The primary outcome of this trial was to measure the change in VAS score from baseline to each observed time-point during the whole phases. The mean change in VAS score after treatment relative to baseline was −4.6 (SD, 0.2) for active EA plus CBZ, −3.0 (SD, 0.2) for sham EA plus CBZ, −3.7 (SD, 0.3) for active EA plus placebo, and −0.9 (SD, 0.3) for sham EA plus placebo (eFigure 3A). The main effects of EA and CBZ were both significant (*P* < 0.001), and there was a significant interaction between the two interventions (*P* = 0.041). Participants who received EA (mean difference [MD], −0.3 [95%CI, −0.40 to −0.20] at week 2; −1.6 [−1.70 to −1.50] at week 4; −1.1 [−1.31 to −0.89] at week 16; −0.8 [−1.01 to −0.59] at week 28), CBZ (MD, −0.6 [95% CI, −0.70 to −0.50] at week 2; −0.9 [−1.03 to −0.77] at week 4, −0.2 [−0.41 to 0.01] at week 16, 0.2 [−0.01 to 0.41] at week 28), and the combination of both (MD, −1.8 [95% CI, −1.90 to −1.70] at week 2; −3.7 [−3.83 to −3.57] at week 4, −3.4 [−3.61 to −3.19] at week 16, −2.9 [−3.11 to −2.69] at week 28) had a larger reduction in VAS score over the treatment course than their respective control groups (sham EA, placebo, and sham EA plus placebo) (Table 2). The trajectory of VAS score at all assessment visits was shown in eFigure 3B.

**Table 2:** Primary outcome analysis of pain intensity in TN patients

Outcomes	EA + CBZ (n = 30)	SEA + CBZ (n = 29)	EA + P (n = 29)	SEA + P (n = 29)
The mean change in VAS score relative to baseline^a^				
Week 2	−2.3 (0.2)	−2.0 (0.2)	−1.7 (0.2)	−0.5 (0.2)
Week 4	−4.6 (0.2)	−3.0 (0.2)	−3.7 (0.3)	−0.9 (0.3)
Week 16	−4.9 (0.4)	−3.8 (0.4)	−4.7 (0.4)	−1.5 (0.4)
Week 28	−4.5 (0.4)	−3.7 (0.4)	−4.7 (0.4)	−1.6 (0.4)
Effect of active EA^b^	Mean Difference (95% CI)			
Week 2	−0.3 [−0.40, −0.20]*			
Week 4	−1.6 [−1.70, −1.50]*			
Week 16	−1.1 [−1.31, −0.89]*			
Week 28	−0.8 [−1.01, −0.59]*			
*P* value for main effect of EA^**c**^	< 0.001			
Effect of CBZ^d^				
Week 2	−0.6 [−0.70, −0.50]*			
Week 4	−0.9 [−1.03, −0.77]*			
Week 16	−0.2 [−0.41, 0.01]			
Week 28	0.2 [−0.01, 0.41]			
*P* value for main effect of CBZ^**e**^	< 0.001			
Effect of EA plus CBZ^f^				
Week 2	−1.8 [−1.90, −1.70]*			
Week 4	−3.7 [−3.83, −3.57]*			
Week 16	−3.4 [−3.61, −3.19]*			
Week 28	−2.9 [−3.11, −2.69]*			
*P* value for EA × CBZ interaction	0.041			
*P* value for main effect of time	< 0.001			
*P* value for EA × CBZ × time 3-way interaction	0.017			

^a^The mean *(SD)* was used to recorded the results of groups, and the change in each outcome was analyzed from baseline to week 28. VAS score range is from 0 to 10, with higher scores indicating greater pain

^b^Repeated-measures ANOVA with factorial design was applied to assess the effect of electroacupuncture, and the *Sidak* test was used for multiple comparisons between groups

^c^Electroacupuncture main effect: (electroacupuncture plus carbarmazepine) + (electroacupuncture plus placebo) vs (sham electroacupuncture plus carbarmazepine) + (sham electroacupuncture plus placebo)

^d^Repeated-measures ANOVA with factorial design was applied to assess the effect of carbarmazepine, and the *Sidak* test was used for multiple comparisons between groups

^e^Carbarmazepine main effect: (electroacupuncture plus carbarmazepine) + (sham electroacupuncture plus carbarmazepine) vs (electroacupuncture plus placebo) + (sham electroacupuncture plus placebo)

^f^Repeated-measures ANOVA with factorial design was applied to assess the efficacy of electroacupuncture combined with carbarmazepine (at the dosage of 300 mg/day), and the *Sidak* test was used for multiple comparisons between groups

*P < 0.05

Abbreviations: *VAS* visual analogue scale, *CI* confidence interval, *EA + CBZ* electroacupuncture plus carbarmazepine (at the dosage of 300 mg/day), *SEA + CBZ* sham electroacupuncture plus carbarmazepine (at the dosage of 300 mg/day), *EA + P* electroacupuncture plus placebo, *SEA + P* sham electroacupuncture plus placebo

### Secondary outcomes

#### Other outcomes to assess pain efficacy

The main effects of EA were significant in PRI, PPI, and the total number of pain attacks (*P* < 0.001), However, the main effect of CBZ was only significant for PRI (*P* = 0.011). A significant interaction was detected between the interventions in PRI (*P* = 0.022), while there were no interactions in PPI and the total number of pain attacks (*P* > 0.05) (Table 3). Additionally, the combination of EA and CBZ was significantly more effective than EA or CBZ alone in reducing the number of pain attacks from baseline to week 2 and week 4 (*P* < 0.05) (eTable 2). Compared to controls, EA (MD, −3.20 [95% CI, −5.24 to −1.15] at week 4) showed a significant decrease in PRI score (MD, −0.64 [95% CI, −1.09 to −0.19] at week 4) and resulted in greater reductions in PPI score (MD, −0.57 [95% CI, −1.02 to −0.12] at week 16) (eFigure 4).

**Table 3:** The secondary outcomes of interaction effects from the full model, including 2- and 3-way interactions between treatment methods and times

Outcomes	Group	*P* value for EA × CBZ × time 3-way interaction^a^	*P* value for EA × CBZ interaction^a^	*P* value for main effect of EA^a^	*P* value for main effect of CBZ^a^	*P* value for main effect of time^a^
Other outcomes to assess efficacy						
SF-MPQ^b^						
PRI	EA + CBZ	0.041*	0.022*	< 0.001*	0.011*	< 0.001*
	SEA + CBZ					
	EA + P					
	SEA + P					
PPI	EA + CBZ	0.026*	0.196	< 0.001*	0.057	< 0.001*
	SEA + CBZ					
	EA + P					
	SEA + P					
The total number of pain attacks^c^	EA + CBZ	0.319	0.065	< 0.001*	0.208	< 0.001*
	SEA + CBZ					
	EA + P					
	SEA + P					
Quality of life assessment						
SF-36	EA + CBZ	0.002*	0.284	0.002*	0.023*	< 0.001*
	SEA + CBZ					
	EA + P					
	SEA + P					
BPI-Facial^d^						
General interference items	EA + CBZ	0.237	0.046*	< 0.001*	0.048*	< 0.001*
	SEA + CBZ					
	EA + P					
	SEA + P					
Facial interference items	EA + CBZ	0.006*	0.189	< 0.001*	0.009*	< 0.001*
	SEA + CBZ					
	EA + P					
	SEA + P					
PGIC^e^	EA + CBZ	0.071	0.025*	< 0.001*	< 0.001*	< 0.001*
	SEA + CBZ					
	EA + P					
	SEA + P					

^a^Multiple comparisons between the groups were compared by using *Sidak* test

^b^Including PRI and PPI. The PRI was comprised of sensory and affective scale, each selected word was scored from 0 (none) to 3 (severe). The total PRI score was obtained by summing the item scores. The PPI range from 0–5, with higher scores indicating greater pain

^c^The total number of pain attacks was calculated by summing the number of pain attacks in the past two weeks prior to the every assessment time-point

^d^Including general interference items and facial interference items. The total score of general interference items was sum of the 7 questions that address pain interference with general activities of daily living (ADL), score range is from 0 to 70, with higher scores indicating greater impact. The total score of facial interference items was sum of the 7 questions that address pain interference with face-specific daily activities, score range is from 0 to 70, with higher scores indicating greater impact

^e^The PGIC evaluated overall health status as perceived by the patient in a seven-point single-item scale ranging from ‘very much worse’ to ‘very much improved’. For descriptive purposes, patients were classified into three categories according to the PGIC score: disease deterioration (0–3 points), stable disease (4 points) or disease improvement (5–7 points) since the initial baseline visit

*P < 0.05

Abbreviations: *VAS* visual analog scale, *SF-MPQ* Short-Form McGill pain questionnaire, *PRI* pain rating index, *PPI* *present pain intensity*, *BPI-Facial* brief pain inventory-facial scale, *SF-36* Short-Form 36 questionnaire, *PGIC* patient global impression of change, *EA + CBZ* electroacupuncture plus carbarmazepine (at the dosage of 300 mg/day), *SEA + CBZ* sham electroacupuncture plus carbarmazepine (at the dosage of 300 mg/day), *EA + P* electroacupuncture plus placebo, *SEA + P* sham electroacupuncture plus placebo

#### Quality of life assessment

As shown in Table 3, the main effects of EA and CBZ were significant in SF-36, “general interference items”, “facial interference items”, and PGIC (*P* < 0.05). Notably, a significant interaction was detected between these two interventions in the scores of “general interference items” and PGIC (*P* < 0.001). Furthermore, compared to the controls, EA resulted in significantly greater reductions in SF-36 (MD, 10.29 [95% CI, 2.72 to 17.85] at week 4), “general interference items” (MD, −8.42 [95% CI, −14.43 to −2.42] at week 4; −7.03 [−12.77 to −1.28] at week 16), “facial interference items” (MD, −11.38 [95% CI, −17.89 to −4.88] at week 4), and PGIC (MD, 0.48 [95% CI, 0.08 to 0.89] at week 2; 1.25 [0.86 to 1.65] at week 4; 0.78 [0.31 to 1.25] at week 16, 0.79 [0.33 to 1.24] at week 28). Compared to placebo, CBZ only showed beneficial effects in “facial interference items” (MD, −8.89 [95% CI, −16.19 to −1.58] at week 2) (eFigure 4). Additionally, the results of the primary and secondary outcomes analyzed by using the last observation carried forward method were shown in Table 4.

**Table 4:** Analysis of primary and secondary outcomes by using last observation carried forward method

Outcomes	Group	Mean ± *SD*^a^					*P*-value for EA × CBZ × time 3-way interaction^g^	*P*-value for EA × CBZ interaction^g^	*P*-value for main effect of EA^g^	*P*-value for main effect of CBZ^g^	*P*-value for main effect of time^g^
		Baseline (n = 120)	Week 2 (n = 120)	Week 4 (n = 120)	Week 16 (n = 120)	Week 28 (n = 120)					
VAS score^b^	EA + CBZ	6.3(1.1)	4.0(1.3)	1.7(1.2)	1.4(1.6)	1.8(1.8)	0.014*	0.034*	< 0.001*	< 0.001*	< 0.001*
	SEA + CBZ	6.5(1.2)	4.5(1.8)	3.5(1.7)	2.7(2.1)	2.8(2.4)					
	EA + P	6.3(1.0)	4.6(1.0)	2.6(1.2)	1.6(1.8)	1.6(1.7)					
	SEA + P	6.0(1.1)	5.5(1.4)	5.1(1.5)	4.6(1.9)	4.5(2.0)					
SF-MPQ^c^											
PRI	EA + CBZ	14.6 (4.8)	6.0 (3.8)	3.7 (3.7)	3.6 (3.6)	3.6 (4.6)	0.044*	0.019*	< 0.001*	0.009*	< 0.001*
	SEA + CBZ	12.8 (5.2)	8.1 (4.9)	6.8 (4.1)	5.4 (4.5)	5.3 (4.5)					
	EA + P	14.8 (6.2)	7.9 (4.3)	4.4 (3.3)	2.9 (4.2)	2.4 (2.9)					
	SEA + P	13.2 (4.8)	10.7 (4.2)	10.5 (4.4)	9.6 (5.9)	9.2 (5.7)					
PPI	EA + CBZ	1.8 (1.3)	1.0 (1.0)	0.5 (0.7)	0.5 (0.7)	0.5 (0.8)	0.046*	0.171	0.001*	0.038*	< 0.001*
	SEA + CBZ	1.6 (1.4)	1.2 (1.2)	1.1 (1.0)	1.1 (1.0)	0.9 (1.1)					
	EA + P	2.2 (1.2)	1.3 (1.0)	0.6 (0.7)	0.4 (0.6)	0.3 (0.5)					
	SEA + P	1.6 (1.2)	1.7 (1.1)	1.7 (1.0)	1.6 (1.0)	1.7 (1.2)					
The total number of pain attacks^d^	EA + CBZ	199.8 (133.6)	121.3 (98.5)	52.8 (52.5)	33.9 (43.6)	48.2 (56.5)	0.334	0.067	0.001*	0.222	< 0.001*
	SEA + CBZ	161.8 (79.5)	108.1 (57.2)	54.9 (38.8)	33.1 (45.7)	27.9 (31.9)					
	EA + P	230.5 (210.0)	147.8 (164.9)	108.9 (107.5)	78.8 (86.0)	76.8 (87.0)					
	SEA + P	225.0 (211.5)	231.9 (286.0)	198.3 (209.5)	166.7 (211.0)	168.9 (214.1)					
BPI-Facial^e^											
General interference items	EA + CBZ	28.3 (14.6)	12.0 (11.9)	6.3 (10.0)	4.8 (8.6)	6.8 (10.5)	0.184	0.058	0.001*	0.046*	< 0.001*
	SEA + CBZ	27.3 (16.3)	16.9 (14.8)	14.6 (12.3)	11.6 (10.7)	11.0 (11.6)					
	EA + P	25.9 (12.9)	15.0 (9.7)	8.6 (8.2)	5.0 (7.6)	4.6 (6.7)					
	SEA + P	28.2 (16.7)	23.2 (14.9)	22.8 (14.7)	21.3 (15.6)	21.5 (16.2)					
Facial interference items	EA + CBZ	29.8 (16.9)	13.7 (9.3)	6.1 (8.3)	8.4 (12.0)	10.1 (13.0)	0.008*	0.136	< 0.001*	0.004*	< 0.001*
	SEA + CBZ	31.4 (16.9)	19.1 (14.9)	17.0 (15.2)	14.7 (15.6)	12.5 (14.2)					
	EA + P	31.9 (14.9)	22.5 (16.5)	12.3 (10.2)	8.0 (11.3)	7.9 (10.7)					
	SEA + P	33.9 (16.3)	28.5 (14.5)	26.4 (14.9)	25.9 (15.5)	25.8 (17.7)					
SF-36	EA + CBZ	112.0 (17.5)	119.3 (16.9)	127.2 (14.3)	127.1 (13.2)	126.1 (14.0)	0.001*	0.309	0.002*	0.022*	< 0.001*
	SEA + CBZ	111.8 (15.4)	114.5 (14.1)	116.8 (13.8)	121.0 (15.2)	122.9 (15.8)					
	EA + P	102.5 (17.8)	117.0 (16.8)	123.5 (16.8)	126.4 (17.4)	127.4 (15.9)					
	SEA + P	111.1 (14.9)	108.6 (12.9)	110.0 (12.7)	110.5 (12.4)	108.8 (11.8)					
PGIC^f^	EA + CBZ	4.1 (0.4)	6.0 (0.9)	6.6 (0.6)	6.3 (0.9)	6.2 (0.9)	0.059	0.023*	< 0.001*	< 0.001*	< 0.001*
	SEA + CBZ	4.0 (0.0)	5.5 (0.7)	5.4 (0.7)	5.5 (0.8)	5.4 (0.8)					
	EA + P	4.0 (0.2)	5.7 (0.9)	6.3 (0.7)	6.0 (0.9)	6.4 (0.9)					
	SEA + P	4.0 (0.2)	4.6 (0.7)	4.7 (1.0)	4.7 (1.0)	4.8 (0.9)					

^**a**^The Mean *(SD)* was used to recorded the results of groups, and the change in each outcome was analyzed from baseline to week 28, including the *P* value for EA × CBZ × time 3-way interaction, and EA × CBZ interaction, and *P* value for main effect of EA, CBZ, and time respectively

^b^VAS score range is from 0 to 10, with higher scores indicating greater pain

^c^Including PRI and PPI. The PRI was comprised of sensory and affective scale, each selected word was scored from 0 (none) to 3 (severe). The total PRI score was obtained by summing the item scores. The PPI range from 0–5, with higher scores indicating greater pain

^d^The total number of pain attacks was calculated by summing the number of pain attacks in the past two weeks prior to the every assessment time-point

^e^Including general interference items and facial interference items. The total score of general interference items was sum of the 7 questions that address pain interference with general activities of daily living (ADL), score range is from 0 to 70, with higher scores indicating greater impact. The total score of facial interference items was sum of the 7 questions that address pain interference with face-specific daily activities, score range is from 0 to 70, with higher scores indicating greater impact

^f^The PGIC evaluated overall health status as perceived by the patient in a seven-point single-item scale ranging from ‘very much worse’ to ‘very much improved’. For descriptive purposes, patients were classified into three categories according to the PGIC score: disease deterioration (0–3 points), stable disease (4 points) or disease improvement (5–7 points) since the initial baseline visit

^g^Multiple comparisons between the groups were compared by using *Sidak* test

^*^P < 0.05

Abbreviations: VAS, visual analog scale; SF-MPQ, Short-Form McGill pain questionnaire; PRI, pain rating index; PPI, present pain intensity; BPI-Facial, brief pain inventory-facial scale; SF-36, Short-Form 36 questionnaire; PGIC, patient global impression of change; SD, standard difference. EA + CBZ, electroacupuncture plus carbarmazepine (at the dosage of 300 mg/day); SEA + CBZ, sham electroacupuncture plus carbarmazepine (at the dosage of 300 mg/day); EA + P, electroacupuncture plus placebo, SEA + P, sham electroacupuncture plus placebo

#### The proportion of patients using rescue medications

The proportion of patients using rescue medications was significantly lower in the EA-plus-CBZ group, sham EA-plus-CBZ group, and EA-plus-placebo group than in the sham-EA-plus-placebo group at follow-up weeks 16 and 28 (Table 5). Notably, compare to controls, the proportion of patients using rescue medications was significantly lower in the EA group at week 28 (*P* = 0.011). Furthermore, the mean daily dosage of rescue medications used in each group during the whole phases was shown in eTable 3.

**Table 5:** Post-hoc analysis of patients using rescue medications among the 4 groups during all phases^a^

	Proportion of participants using rescue medications, n (%)				Pairwise comparison			
	EA + CBZ (n = 30)	SEA + CBZ (n = 29)	EA + P (n = 29)	SEA + P (n = 29)	The effect of EA		The effect of CBZ	
					Z value	*P* value^a^	Z value	*P* value^a^
Baseline	15 (50.00)	15 (50.00)	14(46.67)	17 (56.67)	0.132	0.895	0.132	0.895
Week 2	1 (3.33)	0 (0.0)	1 (3.45)	4 (13.79)	0.992	0.321	0.024	0.981
Week 4	0 (0.0)	0 (0.0)	0 (0.0)	3 (10.34)	0.000	1.000	0.000	1.000
Week 16	9 (30.00)	12 (41.37)	8 (27.59)	20 (68.97)	0.913	0.361	0.205	0.838
Week 28	6 (20.00)	15 (51.72)	7 (24.14)	21 (72.41)	2.544	0.011*	0.383	0.701

^a^The proportions of participants using rescue medications was compared between groups with the *Fisher’s exact* test

*P < 0.05

Abbreviations: *EA + CBZ* electroacupuncture plus carbarmazepine (at the dosage of 300 mg/day), *SEA + CBZ* sham electroacupuncture plus carbarmazepine (at the dosage of 300 mg/day), *EA + P* electroacupuncture plus placebo; *SEA + P* sham electroacupuncture plus placebo

#### Safety, blinding, and compliance

During the whole study phases, 10.17% of patients experienced minor EA-related AEs; while 25.42% (15 out of 59) of participants who received CBZ experienced adverse events. The most commonly reported CBZ-related AEs were “dermatitis/itchiness”, “dizziness/sleepiness”, and palpitations. CBZ-related AEs occurred in 7 (23.3%) participants in the EA-plus-CBZ group and 8 (27.6%) in the sham-EA-plus-CBZ group (Table 6). However, all AEs were minor or moderate, and no special medical intervention was needed. Patients recovered these events and did not withdraw from the trial. The results of blinding evaluation and compliance were also shown in eTable 4.

**Table 6:** AEs evaluation during all phases

	Group				Total, n (%)	*P* value^a^
	EA + CBZ (n = 30)	SEA + CBZ (n = 29)	EA + P (n = 29)	SEA + P (n = 29)		
EA-related AEs, n (%)	3 (10)	NA	3 (10.3)	NA		
Hematoma	3 (10.0)	NA	2 (6.9)	NA		
Tingling sensation	0 (0.0)	NA	1 (3.4)	NA		
CBZ-related AEs, n (%)	7 (23.3)	8 (27.6)	NA	NA		
Dermatitis /itchiness	4 (13.3)	1 (3.4)	NA	NA		
Dizziness /sleepiness	2 (6.7)	4 (13.8)	NA	NA		
Palpitations	1 (3.3)	3 (10.3)	NA	NA		
Total incidence of EA-related AEs, n (%)					6 (10.2)	0.030*
Total incidence of CBZ-related AEs, n (%)					15 (25.4)	

^a^Data were examined using *Chi-square* or *Fisher Exact* test

*P < 0.05

Abbreviations: *NA* not applicable, *EA + CBZ* electroacupuncture plus carbarmazepine (at the dosage of 300 mg/day), *SEA + CBZ* sham electroacupuncture plus carbarmazepine (at the dosage of 300 mg/day), *EA + P* electroacupuncture plus placebo, *SEA + P* sham electroacupuncture plus placebo

## Discussion

This multi-centre, double-blind, randomized trial demonstrated that active EA and CBZ were relatively superior to sham EA and placebo in reducing VAS scores and improving quality of life for patients with TN. Moreover, the combination of EA and low dosage of CBZ (300 mg/day) showed larger benefits than EA or CBZ alone, which therefore may offer benefit to those with TN who cannot tolerate higher dosage of CBZ.

EA is commonly used to treat various pain conditions, including TN. This study further demonstrated that EA can alleviate the pain intensity of TN, with the effects persisting for a minimum of 28 weeks. Moreover, the combination of EA and a low dosage of CBZ was found to have a more pronounced analgesic effect compared to CBZ alone, both immediately after treatment and during the follow-up phase. This may due to the ability of EA to regulate inflammatory mediators, improve the expression of pain-related receptor proteins, and inhibit the generation of negative emotions [32–34]. These findings suggested that a synergistic analgesic effect existed between EA and CBZ, especially EA combined with a low dosage of CBZ (300 mg/day) showing a shorter latency for alleviating pain intensity compared to CBZ alone. Herein, the combination treatment of EA and low dosage of CBZ may be a clinically effective choice for certain patients with TN.

In this study, the total number of pain attacks was focused by the researchers. Specifically, the therapeutic effect of EA for TN was remarkably superior to that of placebo in reducing the number of pain attacks. Previous evidences had demonstrated that EA was effective in reducing both pain severity and the frequency of pain episodes in patients with both migraine and angina [35, 36], which was consistent with our findings in TN. Although the combination of EA and a low dosage of CBZ showed a significant decrease in pain attacks compared to EA or CBZ alone, there was no significant interaction between two treatments. To the best of our knowledge, the nature of pain is subjective, making it difficult to measure quantitatively [37]. Hence, patient-reported outcomes, such as the BPI-facial, were essential in measuring pain relief and improving facial-specific activities after treatment [27]. Additionally, the study also found that EA had a significantly lower impact on “general interference items” and “facial interference items” compared to sham EA, while EA combined with CBZ (300 mg/day) had moderately active effects in improving face-specific activities (such as eating, touching the face, brushing, smiling, and talking) compared to CBZ during the treatment phases.

CBZ is a recommended medication for TN; however, limited to extent due to its AEs. Previous studies had shown that higher dosages of CBZ (more than 600 mg) could increase the higher probability of AEs [15]. As for the correction between the dosage of CBZ and its therapeutic result, a previous study indicated that the change in VAS score was 2.2 after 4 weeks of oral CBZ (at dosage of 300–600 mg/day) [38]. In this study, the change in VAS score was decreased by 3.0 with a daily dosage of 300 mg CBZ, and decreased by 4.6 with EA combined with CBZ, suggesting that a low dosage of CBZ was effective, however, the analgesic effect was significantly improved when combined with EA. Hence, the study supported the use of EA combined with a low dosage of CBZ (at a dosage of 300 mg/day) for TN, to achieve a better analgesic effect and to effectively improve the quality of life in TN patients after treatment relatively to baseline.

Although some reports had indicated that sham-EA interventions, which stimulated superficially at non-acupoints (located adjacent to the traditional acupoints) with no current output, can also considered to induce analgesic effects similar to true EA [39, 40], this study found that EA specifically possessed an analgesic effect in TN. This may be attributed to two factors: (1) the severity of pain reported by patients at baseline may have influenced the therapeutic effects, therefore, only participants with a VAS score greater than 5 were included; and (2) EA was applied without hesitation in this trial due to its advantages in alleviating pain intensity. Previous studies have shown that EA has a faster response time than manual acupuncture, which requires more time to achieve a similar analgesic effect [41]. While there are some acupuncture-related AEs, such as a small hematoma, there were localized at the acupoint and rarely affected normal daily work or quality of life. This phenomenon could be easily avoided with proper acupuncture manipulation. In contrast, CBZ-related AEs, such as “dermatitis/itchiness, dizziness/sleepiness, and palpitations”, were systemic and long-lasting, significantly impacting daily routine and quality of life, and even requiring additional medications to ameliorate symptoms. Therefore, compared to the annoying CBZ-related AEs, hematomas caused by EA recovered quickly without extra treatment and could be avoided with proper manipulation during EA treatment.

### Strengths and limitations of this study

This study has several strengths. First, combination of EA and a low dosage of CBZ was used for the first time, demonstrating the synergistic effect of the combination, and avoiding the serious adverse events associated with higher dosages of CBZ. In addition, the multi-centre RCT used a sound methodology and a standardized acupuncture protocol, providing an alternative and effective option for the treatment of TN in the future. Furthermore, successful blinding was the another strength in this trial. Blinding is an important method for reducing bias in clinical trials, while the blinding of acupuncture is not easy to carry out [42]. The blinding method of non-acupoints without currents output was chosen in sham EA groups in this trial, and the blinding evaluation also indicated that unblinding was prevented. Actually, therapeutic effect of real EA and CBZ may contributed to the higher proportions of correct-guessing in the EA plus CBZ group, while the insertion of needles into non-acupoints without currents output also helped to increase the percentage of participants who believed they received the real combination. Since therapeutic bias will be prevented by a successful blinding, firm belief in being given the active treatment decreased the possibility of participants’ psychological interference. Furthermore, successful blinding also increased the compliance of patients, particularly in those assigned to the sham EA or placebo groups.

However, there were limitations in this study that were unavoidable. Firstly, a fixed EA protocol was used to assess the effectiveness of EA rather than individualized treatment plans based on the acupuncturist’s experience, which may lead to performance bias. However, using a standardized treatment protocol ensured the quality control during the trial. Secondly, although EA combined with low-dosage of CBZ (300 mg) exerted a synergistic effect on TN, there are several issues about the combination of EA and CBZ that remain to be explored in future research studies. For example, the combination of EA and low-dosage CBZ (300 mg) is equally efficacious or less efficacious than high-dosage CBZ (600 mg or more)? The combination of EA and high-dosage CBZ is more efficacious than high-dosage CBZ, etc. Lastly, this trial was initiated in 2017, which lead to the latest version of the diagnostic criteria released in 2018 was not used. However, the findings of this study also provide valuable insights for the treatment of TN.

## Conclusion

EA or CBZ alone are effective treatments against TN, while the combination of EA and low-dosage CBZ exerts a greater benefit. The findings in this trial demonstrate that the combination of EA and low-dosage CBZ may be clinically effective under certain circumstances, especially may offer benefit to those with TN who cannot tolerate higher doses of CBZ.

### Supplementary Information

Below is the link to the electronic supplementary material.

Supplementary file1 (DOC 67826 KB)

## Data Availability

All data generated and analyzed during the current study will be available from the corresponding author on reasonable request.
