# Supplementary material for: Electroacupuncture and carbamazepine for patients with trigeminal neuralgia: a randomized, controlled, 2 × 2 factorial trial
Source: J Neurol. 2024 May 31;271(8):5122–36. doi: 10.1007/s00415-024-12433-x (PMC11319385; doi:10.1007/s00415-024-12433-x)
Supplement: Supplementary file 1 — Supplementary file1 (DOC 67826 KB) [file 415_2024_12433_MOESM1_ESM.doc]

eFigure 1 Study design

eFigure 2 (A) The location of acupoints used in the EA group. (B) The location of non-acupoints used in the sham EA group

eFigure 3 (A) The mean change of VAS score at week 4 relative to baseline (B) The trajectory of the mean change of VAS score during all phases in four groups

eFigure 4 Forest plots of the effects of EA and CBZ on secondary outcomes

eTable 1 Acupoints, stimulation, and location

eTable 2 Analysis of the changes in total number of pain attacks during the phases

eTable 3 Analysis of the mean daily dosage of rescue medications used in each group during the phases

eTable 4 Blinding, and compliance evaluation during the phases


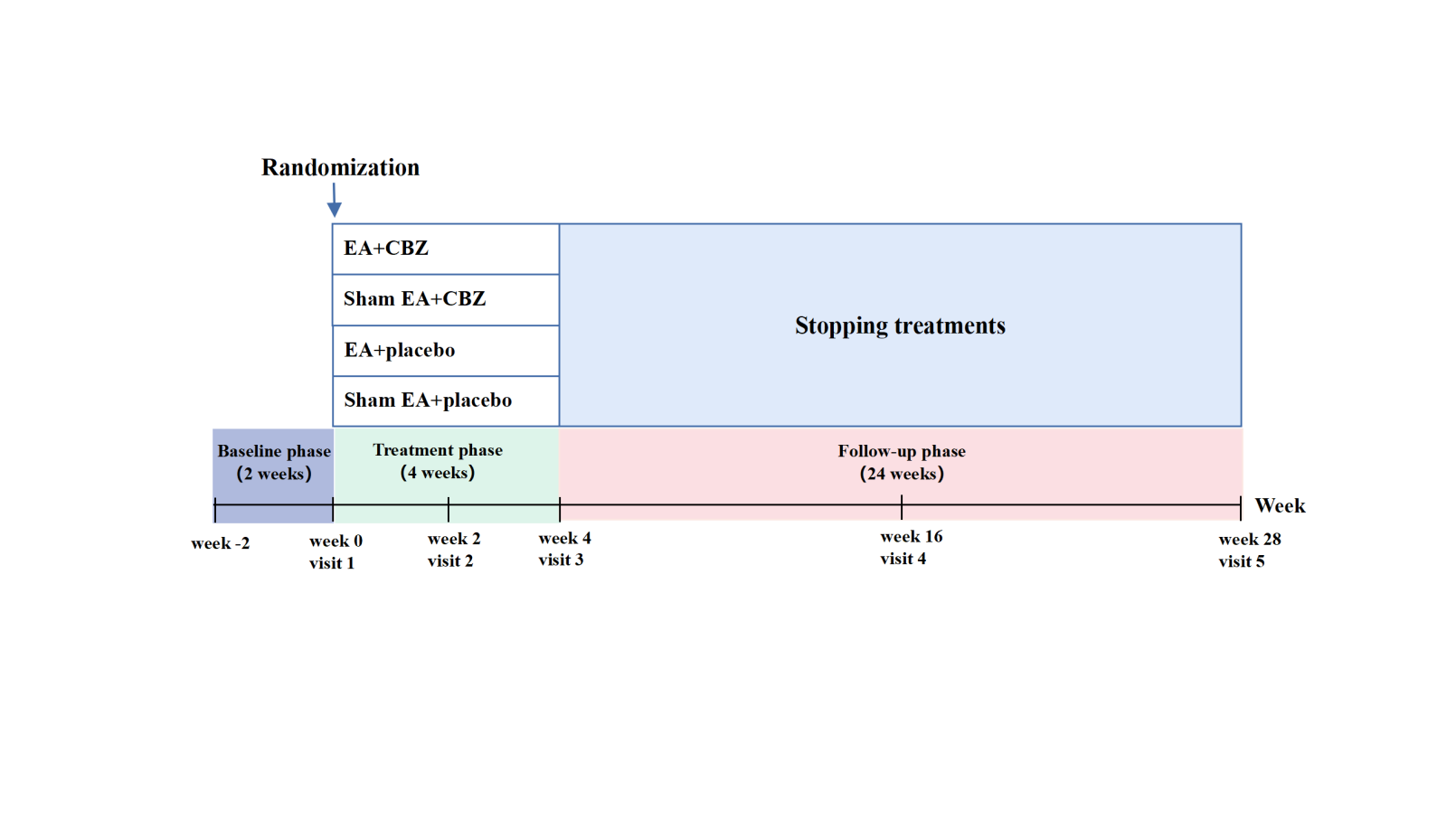


**eFigure 1 Study design**

**Abbreviations:** EA, electroacupuncture; CBZ, carbarmazepine.

**Note:** Rescue medication was allowed throughout the trial (baseline, treatment phase, follow-up phase). During follow up, only rescue medication was allowed to a maximum daily dosage of 600mg. The timing and dosage of all rescue medications was recorded.


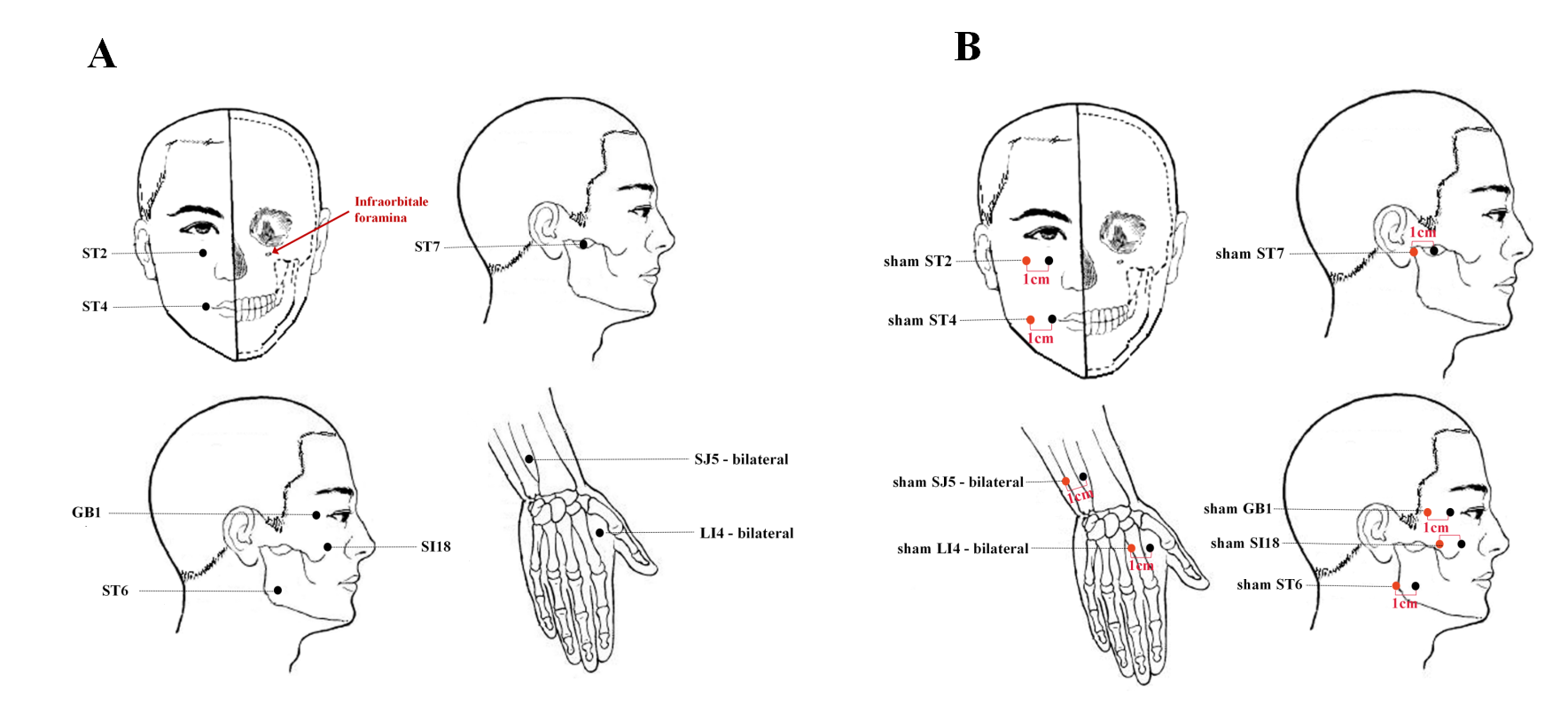


**eFigure 2** **(A) The location of acupoints used in the EA group. (B) The location of non-acupoints used in the sham EA group.** ST2-Sibai; ST4-Dicang; ST7-Xiaguan; GB1-Tongziliao; SI18-Quanliao; ST6-Jiache; SJ5-Waiguan; LI4-Hegu. ST7/GB1, ST7/SI18, or ST7/ST6 were chosen for lesions in the 1^st^ trigeminal division (Ophthalmic branch), the 2^nd^ trigeminal division (Maxillary branch), or the 3^rd^ trigeminal division (Mandibular branch) respectively, which received EA treatment.


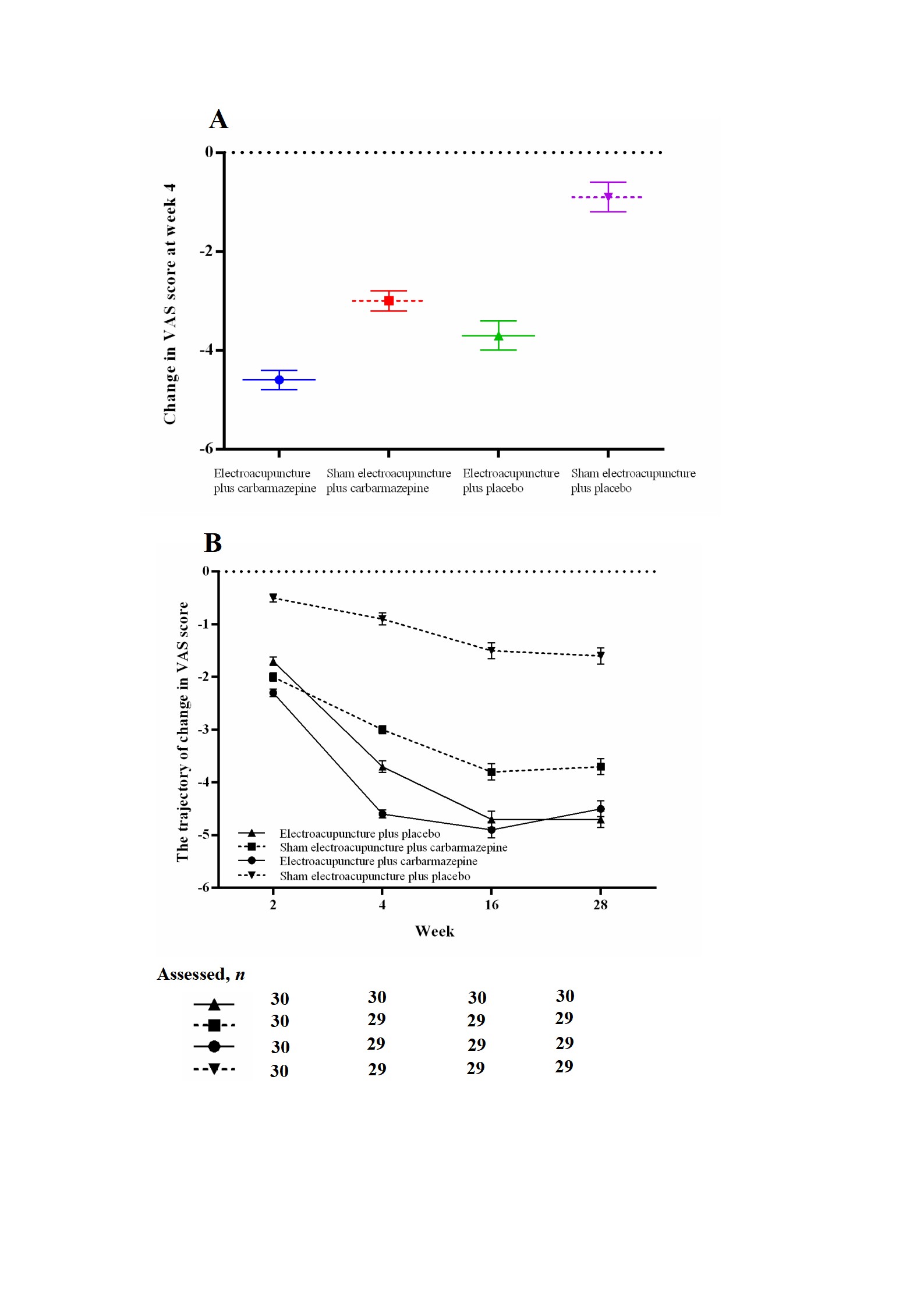


**eFigure 3 (A) The mean change of VAS score at week 4 relative to baseline (B)** **The trajectory of the mean change of VAS score during the all phases in four groups.**

**eFigure 4 Forest plots of the effects of EA and CBZ on secondary outcomes**

**eTable1 Acupoints, stimulation, and location**

| **Acupoints** | | **Stimulation** | **Location** |
| --- | --- | --- | --- |
| **Main** | ST2 | MA | At the infraorbital foramen |
|  | ST4 | MA | About 0.4 cun lateral to the corner of the mouth, directly below the pupil of the eye |
|  | ST7 | EA | Anterior to the ear, in the depression between the zygomatic arch and the condyloid process of the mandible |
| **Localisation of the TN attack** | | |  |
| The 1st trigeminal division | GB1 | EA | 0.5 cun lateral to the outer canthus on the lateral side of the orbit |
| The 2nd trigeminal division | SI18 | EA | Directly below the outer canthus of the eye, in the depression on the lower border of zygoma |
| The 3rd trigeminal division | ST6 | EA | One finger - breadth (middle finger) anterior and superior to the lower angle of the mandible, where masseter attaches, at the prominence of the muscle when teeth are clenched |
| **Distal** | SJ5 | EA | On the dorsal aspect of the forearm, 2 cun above the transverse crease of the wrist between the ulna and radius |
|  | LI4 | EA | On the dorsum of the hand, in the middle of the 2nd metacarpal bone on the radial side |

The location was determined by the People's Republic of China National Standard, “*The Name and Location of Acupoints*” (GB/T 12346-2006); *ST7/GB1*, *ST7/SI18*, or *ST7/ST6* were chosen for lesions in the 1^st^ trigeminal division (ophthalmic branch), the 2^nd^ trigeminal division (maxillary branch), or the 3^rd^ trigeminal division (mandibular branch) respectively, which received EA treatment.

Abbreviations: ST, stomach; GB, gallbladder; SI, small intestine; SJ, sanjiao; LI, large intestine; MA, manual acupuncture; EA, electroacupuncture

| **eTable2** **Analysis of the changes** **in total numbers of pain attacks during the phases** | | | | | | | | | | |
| --- | --- | --- | --- | --- | --- | --- | --- | --- | --- | --- |
|  | **Group^a^** |  |  |  | **The effect of EA^b^** | | | **The effect of CBZ^b^** | | |
| **Outcomes** | **EA+CBZ (n=30)** | **SEA+CBZ (n=29)** | **EA+P (n=29)** | **SEA+P (n=29)** | **Between-group difference** | **95%CI** | ***P* value^c^** | **Between-group difference** | **95%CI** | ***P* value^c^** |
| **The changes in total number of pain attacks^d^** | | | | |  |  |  |  |  |  |
| Week 2 | -78.53 (19.59) | -56.45 (19.93) | -83.31 (19.93) | 6.93 (19.93) | 22.08 (5.15) | [11.78, 32.38] | < 0.0001* | -4.78 (5.15) | [-15.08, 5.52] | 0.3568 |
| Week 4 | -146.97 (19.17) | -109.90 (19.50) | -121.38 (19.50) | -25.69(19.50) | 37.07 (5.04) | [26.99, 47.15] | < 0.0001* | 25.59 (5.04) | [15.51, 35.67] | < 0.0001* |
| Week 16 | -165.87 (24.70) | -131.55 (25.12) | -151.17 (25.12) | -56.62 (25.12) | 34.32 (6.49) | [21.33, 47.31] | < 0.0001* | 14.70 (6.49) | [1.71, 27.69] | 0.0272* |
| Week 28 | -151.60 (25.38) | -136.69 (25.81) | -153.03(25.81) | -55.24 (25.81) | 14.91 (6.67) | [1.56, 28.26] | 0.0292* | -1.43 (6.67) | [-14.78, 11.92] | 0.8309 |
|  |  |  |  |  |  |  |  |  |  |  |

^a^ The Mean *(SD)* was used to recorded the results of groups, and the change in each outcome was analyzed from baseline to weeks 2, 4, 16, and 28. VAS score range is from 0 to 10, with higher scores indicating greater pain.

^b^ Values are adjusted means and 95%CI.

^c^ Repeated-measures ANOVA with factorial design was applied to assess the efficacy of EA combined with CBZ (at the dosage of 300mg/day), and the *Sidak* test was used for multiple comparisons between groups. ^*^*P*<0.05.

^d^ The change in total number of pain attacks indicated that the subtraction difference in total numbers of pain attacks at the observed timepoint compared to baseline.

Abbreviations: CI, confidence interval; EA+CBZ, electroacupuncture plus carbarmazepine (at the dosage of 300mg/day); SEA+CBZ, sham electroacupuncture plus carbarmazepine (at the dosage of 300mg/day); EA+ P, electroacupuncture plus placebo, SEA+P, sham electroacupuncture plus placebo.

| **eTable 3 Analysis of the mean daily dosage of rescue medications used in each group during the phases** | | | | | | | | | |  |  |  |
| --- | --- | --- | --- | --- | --- | --- | --- | --- | --- | --- | --- | --- |
|  | **EA+CBZ (n=30)** | **SEA+CBZ (n=29)** | **EA+P**  **(n=29)** | **SEA+P**  **(n=29)** |  | **EA+CBZ vs. SEA+CBZ** | | **EA+CBZ vs. EA+P** | | **EA+CBZ vs. SEA+P** | | |
|  |  |  |  |  |  | **Between-group difference** | ***P* value** | **Between-group difference** | ***P* value** | **Between-group difference** | | ***P* value** |
| **The mean dosage of CBZ (mg/d)** | | | | |  |  |  |  |  |  | |  |
| Baseline | 236.67(47.82) | 253.45(49.25) | 240.23(49.72) | 265.52(47.15) |  | -16.78 (68.63) | 0.808 | -3.56 (68.96) | 0.959 | -28.85 (67.20) | | 0.669 |
| Week 2 | 6.67(6.67) | 0.00(0.00) | 3.45(3.45) | 28.33(21.02) |  | 6.67 (6.78) | 0.330 | 3.22 (7.58) | 0.673 | -21.66 (21.75) | | 0.323 |
| Week 4 | 0.00(0.00) | 0.00(0.00) | 0.00(0.00) | 31.36 (22.32) |  | 0.00 | >0.999 | 0.00 | >0.999 | -31.36 (21.94) | | 0.158 |
| Week 16 | 54.03(19.04) | 83.91(19.62) | 52.87(21.54) | 143.68(27.44) |  | -29.88 (27.34) | 0.279 | -1.16 (28.75) | 0.968 | -89.65 (33.40) | | 0.009* |
| Week 28 | 43.37 (22.32) | 115.29(25.61) | 70.11(26.85) | 217.24(36.53) |  | 71.92 (33.90) | 0.038* | -26.74 (34.82) | 0.445 | -173.87 (42.49) | | 0.0001* |

Abbreviations: EA+CBZ, electroacupuncture plus carbarmazepine (at the dosage of 300mg/day); SEA+CBZ, sham electroacupuncture plus carbarmazepine (at the dosage of 300mg/day); EA+ P, electroacupuncture plus placebo, SEA+P, sham electroacupuncture plus placebo.

^*^*P*<0.05.

| **eTable 4 Blinding, and compliance evaluation during the phases** | | | | | | |
| --- | --- | --- | --- | --- | --- | --- |
|  | | **Group** | | | | ***P* value** |
|  |  | **EA+CBZ (n=30)** | **SEA+CBZ (n=29)** | **EA+P (n=29)** | **SEA+P (n=29)** |  |
| **Blinding evaluation as the** **distribution of guesses of treatment received, n (%)** ^a^ | | | | | | |
|  | EA+CBZ | 30 (100.0) | 18 (62.1) | 28 (96.6) | 18 (62.1) |  |
|  | sham EA+CBZ | 0 (0.0) | 11 (37.9) | 0 (0.0) | 11 (37.9) |  |
|  | EA+placebo | 0 (0.0) | 0 (0.0) | 1 (3.4) | 0 (0.0) |  |
|  | sham EA+placebo | 0 (0.0) | 0 (0.0) | 0 (0.0) | 0 (0.0) |  |
| **Compliance evaluation ^b^, n (%)** | | | | | | 0.795 |
|  | Good | 30 (100.0) | 29 (96.7) | 29 (96.7) | 29 (96.7) |  |
|  | Poor | 0 (0.0) | 1 (3.3) | 1 (3.3) | 1 (3.3) |  |

^a^ All participants were asked to guess the treatment which they have received at the end of the trial and the answers were recorded, which presented as the distribution of guesses of treatment received.

^b^ Total number of treatments was 12 sessions, the compliance rate = (actual treatment times /12)×100%. If the compliance rate is greater than 80%, it means that the compliance rate is excellent.

EA+CBZ, electroacupuncture plus carbarmazepine (at the dosage of 300mg/day); SEA+CBZ, sham electroacupuncture plus carbarmazepine (at the dosage of 300mg/day); EA+ P, electroacupuncture plus placebo, SEA+P, sham electroacupuncture plus placebo.
